# Characterisation of the anti-apoptotic function of survivin-ΔEx3 during TNF*α*−mediated cell death

**DOI:** 10.1038/sj.bjc.6603768

**Published:** 2007-05-15

**Authors:** M-H Malcles, H-W Wang, A Koumi, Y-H Tsai, M Yu, A Godfrey, C Boshoff

**Affiliations:** 1 Cancer Research UK Viral Oncology Group, Wolfson Institute for Biomedical Research, University College, London, UK; 2 Institute of Microbiology and Immunology, National Yang-Ming University, Taiwan; 3 Department of Education and Research, Taipei City Hospital, Taiwan

**Keywords:** apoptosis, Bcl-2, caspase-3, survivin-ΔEx3

## Abstract

Survivin is an oncogenic protein involved in cell division and acts as an anti-apoptotic factor. It is highly expressed in most cancers and is associated with chemotherapy resistance, increased tumour recurrence, and shorter patient survival. This makes anti-survivin therapy an attractive cancer treatment strategy. These functions are mediated by several survivin spliced variants, whose expression may correlate with cancer progression. One of the spliced variants, survivin-ΔEx3, is known to inhibit apoptosis, through undefined mechanisms. Here, we characterised these mechanisms upon TNF*α*−mediated apoptosis, and showed that survivin-ΔEx3 acts as an adaptor, allowing the formation of a complex between Bcl-2 and activated caspase-3. The Bcl-2/survivin-ΔEx3 complex, but not survivin-ΔEx3 itself, inhibits the activity of caspase-3. Bcl-2 is therefore linked to the postmitochondrial apoptotic machinery by survivin-ΔEx3. Thus, survivin-ΔEx3 plays a key role in the inhibition of caspase-3 activity, and in the control of the mitochondrial checkpoint of apoptosis. This study suggests that targeting survivin-ΔEx3, rather than survivin alone, could be relevant for treating human cancers.

Survivin is an inhibitor of apoptosis, which also plays a critical role in regulating cell cycle and mitosis (Altieri, 2004). This protein is highly expressed in most cancers and is associated with tumour cell resistance to apoptotic stimuli. This ability is essential during tumourigenesis by providing cancer cells growth advantages and conferring resistance to chemotherapy. Survivin expression is also associated with tumour recurrence and shorter survival (Altieri, 2001; Reed, 2002).

Regulation of caspase activity is one of the mechanisms used by cancer cells to become resistant to apoptosis (Hengartner, 2000). This function can be achieved by the cytoplasmic inhibitor-of-apoptosis proteins (IAPs). IAPs contain BIR (baculovirus IAP repeat) domains, which are essential for their anti-apoptotic properties. In several cases, BIR domains directly bind to and inhibit caspase activities (Deveraux and Reed, 1999; Sun *et al*, 1999; Goyal, 2001). Caspase activities are also regulated by another anti-apoptotic family: the Bcl-2 family (Frade and Michaelidis, 1997). In mammalian cells, several adaptor proteins bridge caspases to Bcl-2 or Bcl-xL, to control their activation (Ng *et al*, 1997; Chau *et al*, 2000; Zhang *et al*, 2003). Such adaptors link the mitochondrial Bcl-2 family members to the postmitochondrial apoptotic machinery, and thereby play an important role in the regulation of apoptosis.

Survivin was originally identified by its structural homology to the IAP family of proteins in human B-cell lymphoma (Ambrosini *et al*, 1997). In human cells, there are four spliced variants of survivin: survivin-2B; survivin-2*α*, survivin-3B, and survivin-ΔEx3. Their expression levels correlate with cancer progression (Li, 2005). Survivin-ΔEx3 is generated by the removal of exon 3 (Mahotka *et al*, 1999; Badran *et al*, 2004; Caldas *et al*, 2005). The open reading frame of survivin-ΔEx3 encodes a protein with an interrupted BIR domain and a unique 63 amino-acid long C-terminal tail (Mahotka *et al*, 1999). Overexpression of survivin-ΔEx3 is observed in several human malignancies, including renal cell carcinoma, breast cancer, gastric carcinoma, and medulloblastoma (Krieg *et al*, 2002; Mahotka *et al*, 2002a; Fangusaro *et al*, 2005; Ryan *et al*, 2005). In addition, expression of survivin and survivin-ΔEx3 remains constant in different stages of cancer (Krieg *et al*, 2002). Patients with soft-tissue sarcoma also have an increased risk of tumour-related death when survivin-ΔEx3 is overexpressed (Taubert *et al*, 2005). The expression level of survivin-ΔEx3 is inversely correlated with apoptotic index in gastric cancers (Meng *et al*, 2004).

Survivin-ΔEx3 inhibits apoptosis, through undefined mechanisms (Mahotka *et al*, 1999, 2002a). The protein has a unique C-terminus, and novel anti-apoptotic features could be mediated by this region. We previously suggested that a mitochondrial targeting signal (MTS) and a putative BH2 domain may be located in this region (Wang *et al*, 2002). Survivin-ΔEx3 preferentially localises in the nucleus during late G1 to G2 phases of the cell cycle (Fortugno *et al*, 2002). A nuclear localisation signal (NLS) is embedded in its unique C terminus (Mahotka *et al*, 2002b). Survivin-ΔEx3 is also distributed in the cytosol, with a fraction located at the mitochondria in HeLa and Daoy cells (Rodriguez *et al*, 2002; Mahotka *et al*, 2002b; Caldas *et al*, 2005; You *et al*, 2006). Survivin and survivin-ΔEx3 form heterodimers, thereby regulating the balance between proliferation and cell death (Caldas *et al*, 2005). Other binding partners may additionally contribute to the anti-apoptotic function of survivin-ΔEx3.

Previously, we showed that a viral protein vIAP (viral inhibitor-of-apoptosis protein), which is encoded by ORF K7 of human Kaposi's sarcoma-associated herpesvirus (KSHV), is an adaptor between Bcl-2 and activated caspase-3, thereby, enabling Bcl-2 to inhibit caspase-3 activity (Wang *et al*, 2002). We found that vIAP is structurally and functionally related to survivin-ΔEx3: both proteins contain a disrupted BIR domain, an MTS, and a putative BH2 domain (Wang *et al*, 2002). However, it is not yet clear, whether survivin-ΔEx3 also achieves its anti-apoptotic function in a similar way to that of vIAP. We hypothesised that survivin-ΔEx3 may achieve its anti-apoptotic function by bridging mitochondrial proteins, such as Bcl-2, to caspases.

Here, we showed that upon TNF*α* treatment, survivin-ΔEx3 localises at the mitochondria, where it binds to Bcl-2 and to activated caspase-3, acting as an adaptor, which allows Bcl-2 to inhibit the activity of caspase-3. Thus, this study suggests that survivin-ΔEx3 is a central regulator at the mitochondrial checkpoint during TNF*α*-induced apoptosis.

## MATERIALS AND METHODS

### Plasmids

pCR3.1-survivin and pCR3.1-survivin-ΔEx3 expressing haemagglutinin-tagged forms were described previously (Wang *et al*, 2002). pCR3.1-survivin-ΔEx3(ΔBIR) (deleted aa 38–43) and pCR3.1-survivin-ΔEx3(ΔBH2) (deleted aa 101–107) were created using the QuickChange mutagenesis kit (Stratagene, Amsterdam, The Netherlands) to introduce deletion mutations in pCR3.1-survivin-ΔEx3. pGST-survivin-ΔEx3(118), pGST-survivin-ΔEx3(100), pGST-survivin-ΔEx3(70), and pGST-survivin-ΔEx3(CT) were created by PCR amplification from pCR3.1-survivin-ΔEx3, and then cloned in pGEX6P1 vector (Amersham Pharmacia Biotech, Bucks, UK).

### RNA interference

Targets for RNA interference were selected using the Dharmacon sequence selection tool (www.dharmacon.com). First, we attempted to develop a survivin-ΔEx3-specific siRNA. Since survivin contains all the sequences that survivin-ΔEx3 has, we designed siRNA using the only specific sequences of survivin-ΔEx3 at the junction of exons 2 and 4, but we did not obtain any knock-down effect. In consequence, a global approach to knock-down survivin-ΔEx3 was used. DNA oligos containing the target sequence, a TTCG hairpin, the antisense of the target, a five T termination sequence, and a CTAG (*Xba*I site) were synthesised by (MWG Biotech, London, UK), annealed and inserted into the pGEM-U6M plasmid by digestion with *Xba*I and *Sma*I (Promega, Southampton, UK) and ligated with T4 DNA ligase (NEB, Herts, UK). pGEM-U6M was created from pGEM-U6L and altering the +1 base pair of the U6 promoter from G to C using the Stratagene quickchange site directed mutagenesis kit. Lentiviral RNA interference plasmids were then generated by subcloning the U6 promoter-hairpin construct from pGEM-U6M into pCSGW by digestion with *Eco*RI. The short hairpin targeting all three survivin isoforms is ggaccaccgcatctctacattc.

### Glutathione *S*-transferase (GST) pull-down, immunoprecipitation, Western blot, and immunofluorescence microscopy

The expression of recombinant glutathione *S*-transferase (GST) fusion proteins, GST pull-down assays, *in vivo* co-immunoprecipitations, and Western blots were described previously (Wang *et al*, 2002). The following primary antibodies were used: anti-survivin (6E4 mAb, Cell Signaling, Danvers, USA), anti-HA (BabCo, Cambridge, MA, USA), anti-cytochrome *c* (Upstate, Chandlers Ford, UK), anti-Bcl-2 (BD Pharmingen, Oxford, UK), anti-caspase-3 (BD Pharmingen). Transfections were performed using fuGENE6 transfection reagent (Roche, Welwyn Garden City, UK). For immunofluorescent assay (IFA), HeLa cells were fixed and permeabilised using formalin 3.7% and PBS-T-0.1% Triton X-100, as previously described (Wang *et al*, 2002). Images were taken using a confocal microscope (Leica TSC Systems, Bucks, UK).

### Subcellular fractionations

A method described previously (Wang *et al*, 2002) was used to divide cells into intact nucleus, intact mitochondria, and cytosol fractions

### RNA extraction and RT–PCR

RNA extraction and RT–PCR were performed, as previously described (Wang *et al*, 2002). The sense primer for specific amplification of survivin-ΔEx3 cDNA is as follows: 5′-GACGACCCCATGCAAAGGAAAC-3′, and the antisense primer is the same as that used for survivin-ΔEx3/survivin cloning.

### Comparative protein modelling and bioinformatics tools

The MODELLER program (http://salilab.org/modeller/modeller.html) was applied in the comparative modelling work (Sanchez and Sali, 2000). Protein templates used in comparative modelling were downloaded from the PDB database (http://www.rcsb.org/pdb/index.html). The RasMol software (www.umass.edu) and the Swiss-PdbViewer program (v3.7) (www.expasy.org/spdbv) were used to view and analyse the resulting models. To further characterise the intracellular localisation of different proteins, we used the TMprep program (www.ch.embnet.org/software/TMPRED_form.html), which makes a prediction of membrane-spanning regions and their orientation based on the statistical analysis of the TMbase database (www.ch.embnet.org/software/tmbase/TMBASE_doc.html). Another independent algorithm, the TopPrep program (bioweb.pasteur.fr/seqanal/interfaces/toppred.html), is also used to confirm the reliability of the TMprep program.

### Induction of apoptosis, apoptotic assays, and flow cytometry analyses

For TNF*α*-induced apoptosis, transfected HeLa cells were cultured in the presence of 10 ng ml^−1^ of TNF*α* (Sigma, Gillingham, UK) plus 1 *μ*g ml^−1^ of cycloheximide (Sigma) for the indicated time period. For Bax-induced apoptosis, 4 × 10^5^ of cells were transfected with Bax-expression plasmids and incubated at 37°C for 48 h or 24 h. To measure mitochondrial transmembrane potential (Δ*Ψ*m), 5 × 10^5^ of transfected cells were incubated with 500 *μ*M of CMXRosamine (Molecular Probes, Paisley, UK) at 37°C for 30 min. To evaluate cell viability, cells were resuspended in propidium iodide (PI) solution (2 *μ*g ml^−1^ PI in PBS with 1% BSA and 0.01% azide, BD Pharmingen, UK) at 20°C for 20 min. Treated cells were washed in PBS, then analysed for fluorescence using FACSCalibur™ flow cytometry with the CellQuest software (Becton Dickinson, Franklin Lakes, NJ, USA). A total of 20 000 cells were counted in each experiment, and the numbers reported represent the average and standard deviation (s.d.) of at least three independent experiments. For the caspase-3 activity assays, we used the ApoAlert caspase-3 fluorescent assay kit (Clontech, Saint-Germain-en-Laye, France).

## RESULTS

### Expression of survivin-ΔEx3 in human cancer and normal cell lines

**Figure 1:**
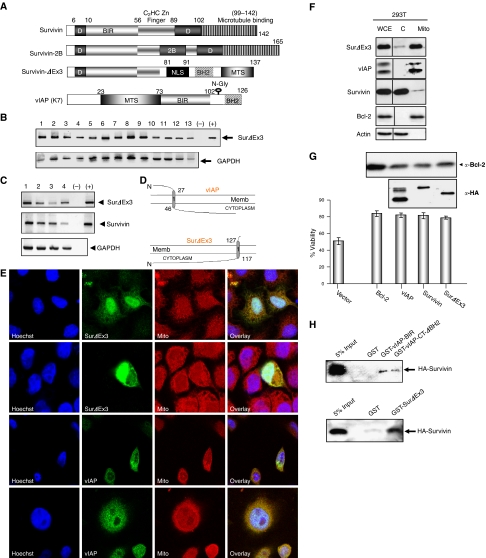
Structure–function relationships of survivin-ΔEx3 and KSHV vIAP protein (vIAP). (**A**) Domain organisation of survivin, survivin-2B, survivin-ΔEx3, and vIAP. The discrete domains (D), involved in dimerisation, BIR and BH2-like domains, the zinc-finger motif, the microtubule binding, the nuclear localisation signal (NLS), mitochondrial targeting signal (MTS) and N-glycosylation (N-Gly) signal are shown. (**B**) Reverse transcription-PCR (RT–PCR) detection of survivin-ΔEx3 transcripts in different cell lines. Lane 1: 293T; lane 2: KS Y-1; lane 3: KS-IMM; lane 4: HeLa; lane 5: HT1080; lane 6: A549; lane 7: H1299; lane 8: Saos2; lane 9: U2OS; lane 10: 1E7; lane 11: ECV304; lane 12: HE-1; lane 13: THP1 cells. (+) cDNA of survivin-ΔEx3 was used as a positive control. (−) water-only negative control. (**C**) RT–PCR detection of survivin-ΔEx3 transcripts in human primary cells. Lane 1: HUVEC; lane 2: MSC; lane 3: DMVEC; lane 4: adult bone marrow mononuclear cells. (**D**) Schematic representation of the transmembrane potential orientations of survivin-ΔEx3 and vIAP (TopPrep program). (**E**) Immunofluorescent assays on cells expressing HA-tagged survivin-ΔEx3 or vIAP. Green: SurΔEx3 or vIAP, blue: DNA stained with Hoechst, red: mitochondria stained with MitoTracker dye. The overlay is presented on the right panel. (**F**) Subcellular fractionation of 293T cells transfected with survivin-ΔEx3, survivin, and HA-vIAP. Transfected cells were separated into cytoplasmic (c) and mitochondrial (m) fractions and analysed by Western blot with anti-survivin-ΔEx3, -HA, and -Bcl-2 antibodies. (**G**) Survivin-ΔEx3 rescues cells from TNF*α*-induced apoptosis. 48 h after transfection, HeLa cells were treated with TNF*α* (10 ng ml^−1^) plus cycloheximide (1 *μ*g ml^−1^) for 2 h, pooled, washed and stained with propidium iodide (PI) to assess cell viability and counted by flow cytometry. Results represent the mean±standard deviation of four independent experiments. (**H**) Both survivin-ΔEx3 and vIAP bind to survivin *in vitro*. The indicated GST-fusion proteins on glutathione beads were incubated with HA-tagged survivin-expressing 293T lysates, and specifically bound proteins were analysed by Western blot with an anti-HA.

The domain organisation of survivin, survivin-2B, survivin-ΔEx3, and vIAP is represented in Figure 1A. We investigated the transcriptional expression of survivin and survivin-ΔEx3 in several human cancer and normal cell lines. The mRNA of survivin-ΔEx3 is detected by semiquantitative RT–PCR in cell lines derived from human cancers, like for Kaposi sarcoma (lanes 2, 3), cervival carcinoma (lane 4), fibrosarcoma (lane 5), non-small lung carcinoma (lane 7), osteosarcoma (lanes 8–9), leukaemia (lane 13), and also from cell lines established from normal tissues, like embryonic kidney cells (lane 1), pulmonary epithelial cells (lane 6), endothelial cells from umbilical vein (lanes 10, 11). All the cell lines tested were positive for survivin-ΔEx3 (Figure 1B). Expression of both survivin and survivin-ΔEx3 transcripts has also been detected in human primary cells (Figure 1C). Survivin-ΔEx3, like survivin (Adida *et al*, 1998), is detectable in fetal tissues, suggesting that this protein may be another oncofetal protein deregulated in cancers.

### Localisation of survivin-ΔEx3 and vIAP

We next analysed the sequence of survivin, survivin-ΔEx3, and vIAP, using computational protein modelling tools. Two different computational analyses suggested a putative localisation to the membrane of survivin-ΔEx3, with inside-out orientation of the C-terminus domain (Figure 1D).

We next characterised the localisation of survivin-ΔEx3 and vIAP using IFA in survivin-ΔEx3- or vIAP-expressing HeLa cells, stained with a mitochondria-specific dye (Figure 1E). Consistent with previous reports (Caldas *et al*, 2005; You *et al*, 2006), survivin-ΔEx3 and vIAP are localised in the nucleus, the cytosol, and particularly in the mitochondria (Figure 1E). Using subcellular fractionation assays, we showed that both proteins can be found in the mitochondrial fraction in HeLa cells. The mitochondrial distribution pattern of survivin-ΔEx3 and vIAP was similar to that of Bcl-2, but distinct from that of survivin (Figure 1F), which is known to reside in the cytoplasm and to translocate into the nucleus during mitosis (Fortugno *et al*, 2002).

### Survivin-ΔEx3 protects cells from apoptosis

We tested if survivin-ΔEx3 shares with vIAP its ability to protect cells from apoptosis, upon different apoptotic treatments. We investigated the mitochondrial function of survivin-ΔEx3, and showed that the expression of survivin-ΔEx3 prevents cytochrome *c* release in 293 cells upon Bax-induced apoptosis (Figure 3C) and protects HeLa cells from TNF*α*-induced apoptosis (Figure 1G). The ability of survivin-ΔEx3 to protect cells from apoptosis was comparable to that of KSHV vIAP, survivin, and Bcl-2. It was reported that the diverse functions of survivin may be explained partly by its ability to heterodimerise with its splicing variants in tumour cells (Caldas *et al*, 2005). Survivin and survivin-ΔEx3 interact at the mitochondria, where they may protect cells from mitochondrial-dependent apoptosis (Caldas *et al*, 2005). Using a GST pull-down, we further showed that both vIAP and survivin-ΔEx3 could heterodimerise with survivin, via the BIR domain (Figure 1H).

### Survivin-ΔEx3 associates with both Bcl-2 and activated caspase-3

**Figure 2:**
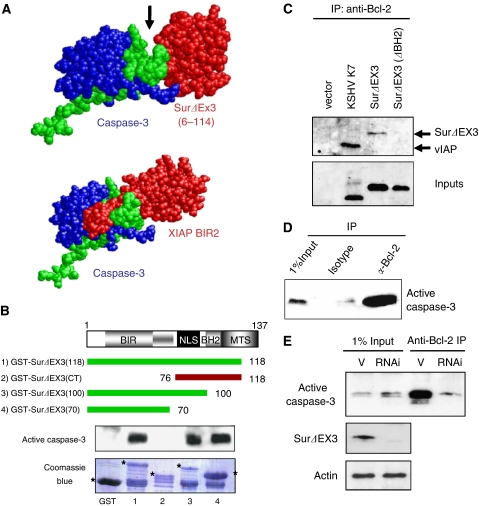
Survivin-ΔEx3 functions as an adaptor. (**A**) A predicted model for survivin-ΔEx3 in complex with active caspase-3. Caspase-3 catalytic domain (upper panel) is composed of a large (blue) and a small (green) subunit, and binds to survivin-ΔEx3 BIR domain (red). The substrate-binding pocket is shown with an arrow. This model is based on the existing interaction between active caspase-3 and the XIAP BIR2 domain (lower panel). (**B**) Survivin-ΔEx3 interacts directly through its BIR domain with active caspase-3. Upper, schematic representation of the domain organisation of the recombinant proteins. Green lines indicate caspase-3-binding proteins, while red does not. Lower, bound caspase-3 is analysed by Western blot with an anti-active caspase-3 polyclonal antibody. A Coomassie blue-stained gel shows the expression level of the different mutants. (**C**) Survivin-ΔEx3 binds to Bcl-2 *in vivo*, through its BH2 domain. Immunoprecipitations were performed in HeLa transfected with indicated plasmids. (**D**) Bcl-2 precipitates active caspase-3 in the presence of survivin-ΔEx3. Survivin-ΔEx3-expressing HeLa cells were treated with TNF*α* (10 ng ml^−1^) plus cycloheximide (1 *μ*g ml^−1^) for 2 h. Immunoprecipitations were performed as previously, and were analysed with anti-active caspase-3 antibody. (**E**) Survivin-ΔEx3 is an essential bridge between Bcl-2 and active caspase-3. HeLa cells were pre-infected with either empty lentivirus (V) or lentivirus stably expressing RNAi against all isoforms of survivin (KO). Apoptosis and immunoprecipitation were performed as described previously.

Next, we investigated the mechanisms by which survivin-ΔEx3 inhibits TNF*α*-mediated apoptosis. Certain mammalian IAPs and the KSHV vIAP are able to bind directly to caspases, via their BIR domains (Deveraux and Reed, 1999; Goyal, 2001; Wang *et al*, 2002). We therefore investigated whether the disrupted BIR domain of survivin-ΔEx3 could still bind to the active form of caspase-3. To gain further insights into the interaction between activated caspase-3 and survivin-ΔEx3, we computationally modelled the putative survivin-ΔEx3–caspase-3 complex. According to the published structure of the XIAP BIR2-caspase-3 complex (PDB ID: 1I3O (Riedl *et al*, 2001), Figure 2A, lower panel), the topological contact between survivin-ΔEx3 and active caspase-3 is predicted to be through the globular BIR domain of survivin-ΔEx3 with the edge, but not with the catalytic pocket of the enzyme caspase-3 (Figure 2A, upper panel). In contrast, it is reported that XIAP can further bind to the substrate-binding cleft of active caspase-3, via its N-terminal linker, hence, providing a steric blockade mechanism for substrate binding (Figure 2A, lower panel). This computational model implied that although survivin-ΔEx3 could bind to active caspase-3, this interaction may not be sufficient for survivin-ΔEx3 to inhibit the activity of this enzyme. Other cofactors may be required for the anti-apoptotic function of survivin-ΔEx3.

We tested if survivin-ΔEx3 can interact directly with activated caspase-3. Four different deletion mutants of survivin-ΔEx3 were designed and cloned in fusion with recombinant GST to map the different domains within survivin-ΔEx3 potentially involved in the interaction with caspase-3 (Figure 2B, upper panel), and a GST pull-down assay was performed. We showed that recombinant active caspase-3 is pulled down by survivin-ΔEx3 and that only the interrupted BIR domain is necessary and sufficient for this interaction (Figure 2B, lanes 1 and 4). These interactions were specific, as GST only, or the C-terminus of survivin-ΔEx3, which does not contain BIR domain did not pull down active caspase-3 (Figure 2B, lanes GST and 2).

We have previously shown that the KSHV vIAP suppresses caspase-3 activity, only when it also binds to Bcl-2 (Wang *et al*, 2002). It is possible that survivin-ΔEx3 also inhibits indirectly caspase-3 activity, by also binding to Bcl-2. We therefore tested survivin-ΔEx3 and Bcl-2 interaction by *in vivo* co-immunoprecipitation (IP) in HeLa cell extracts expressing vIAP, survivin-ΔEx3, and survivin-ΔEx3(ΔBH2) with an anti-Bcl-2 mAb. We showed that survivin-ΔEx3 and vIAP (used here as a positive control), but not the BH2 domain-deleted survivin-ΔEx3 mutant, were immunoprecipitated with endogenous Bcl-2 (Figure 2C). This experiment showed that survivin-ΔEx3 binds to Bcl-2 via its BH2 domain.

To test the interaction between Bcl-2 and activated caspase-3, an *in vivo* Bcl-2 IP was performed in a TNF*α*-treated HeLa cell lysate expressing endogenous survivin-ΔEx3. The active form of caspase-3 was immunoprecipitated with Bcl2, validating the existence of a tripartite complex between survivin-ΔEx3, Bcl-2, and active caspase-3 (Figure 2D).

The ability of survivin-ΔEx3 to mediate the interaction between caspase-3 and Bcl-2 was tested by knocking down endogenous survivin-ΔEx3. Short hairpin RNAs expressed by a lentiviral vector were used to eliminate the expression of all endogenous survivin isoforms, and an *in vivo* Bcl2 IP was performed on survivin-ΔEx3 positive- and negative-HeLa cell lysates. Activated caspase-3 could be immunoprecipitated in complex with Bcl-2, only when survivin-ΔEx3 is present (Figure 2E). The absence of caspase-3 on Bcl-2 IP when all isoforms of survivin are knocked down is likely to be due to the absence of survivin-ΔEx3, rather than survivin, as survivin does not interact with Bcl-2 (Supplementary Figure B).

### Survivin-ΔEx3 associates with Bcl-2 to inhibit the function of activated caspase-3

**Figure 3:**
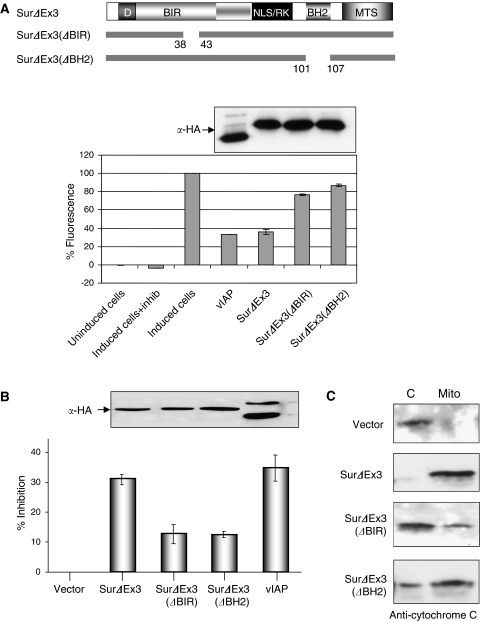
Functional analysis. (**A**) Requirement of both survivin-ΔEx3 BH2 and BIR domains for caspase-3-inhibition. At 48 h after transfection of the indicated plasmids, and after 2 h of TNFα/CHX treatment, fluorescent substrate (Sub) and cellular extracts expressing the different constructs were mixed together and reactions were incubated for 1 h at 37°C, before monitoring fluorescence on a fluoremeter. Results present caspase-3 activity in per cent, each sample standardised to the noninduced cellular extracts, as requested by the manufacturer. (**B**) Both BH2 and BIR domains of survivin-ΔEx3 are essential for its anti-apoptotic function. At 48 h after transfection, in HeLa cells transfected with the indicated expression constructs and exposed to TNF*α*/cycloheximide during 2 h, MitoTracker was used to measure the loss of mitochondrial membrane potential (Δ*Ψ*m). Inhibition percentage was calculated as follows: (% apoptosis in vector-transfected cells)−(% apoptosis in the indicated DNA-transfected cells)/(% apoptosis in vector-transfected cells), where % apoptosis is the percentage of apoptotic cells relative to total cells. (**C**) Survivin-ΔEx3 can inhibit Bax-induced cytochrome *c* translocation. Subcellular fractionation assays were performed with 293T cells expressing the different constructs. Cytosolic fraction (**C**) and mitochondrial fractions (Mito) were blotted with an anti-cytochrome *c* antibody.

The functional significance of the association between survivin-ΔEx3, Bcl-2, and active caspase-3 was further addressed by testing the ability of survivin-ΔEx3 and several mutants to inhibit endogenous caspase-3 activity upon TNF*α*-induced apoptosis, using a caspase fluorescent substrate assay. We found that survivin-ΔEx3, like vIAP (Wang *et al*, 2002), inhibits caspase-3 activity. Such inhibition required the presence of a functional BH2 domain (necessary for binding to Bcl-2) and also a functional BIR domain (necessary for binding to caspase-3; Figure 3A). Using recombinant caspase-3 also showed that both BH2 and BIR domains are required (Supplementary Figure A). Overall, these data indicate that survivin-ΔEx3 protects cells from TNFα-induced apoptosis, by acting through a caspase-3-dependent pathway.

Finally, we investigated whether the ability of survivin-ΔEx3 to associate to Bcl-2 and caspase-3 is essential for its anti-apoptotic function. The BH2 and BIR domains of survivin-ΔEx3 were deleted, and we monitored for each mutant their aptitude to protect cells by measuring the loss of mitochondrial membrane potential (Δ*ψ*m), using a dye specific for intact mitochondrial membranes (Figure 3B). Survivin-ΔEx3 protects HeLa cells from TNF*α*-induced apoptosis as much as vIAP, used here as a positive control (Figure 3B). The deletion of the BH2 or of the BIR domains resulted in a significant reduction of the anti-apoptotic activity of survivin-ΔEx3 (Figure 3B).

We also monitored the translocation of cytochrome *c* from mitochondria into the cytosol, as a marker for mitochondrial integrity, upon Bax-induced apoptosis in 293T cells. Survivin-ΔEx3 prevented cytochrome *c* translocation, while the two mutants did not (Figure 3C). The different protective effects of survivin-ΔEx3 mutants cannot be attributed to variations in protein expression level, since all mutants were expressed similarly (Figure 3, Western blot), and all survivin-ΔEx3 remained localised at the mitochondria. Overall, these data suggest that binding to active caspase-3 alone or Bcl-2 alone is not sufficient for survivin-ΔEx3 to inhibit the enzymatic activity of caspase-3. To achieve a complete anti-apoptotic function, survivin-ΔEx3 requires an association with both partners (Figure 3).

## DISCUSSION

We have shown that survivin-ΔEx3 shares a number of similarities with vIAP (Figure 1; Wang *et al*, 2002), such as their localisation to the mitochondrial compartment, their ability to heterodimerise with survivin, and their anti-apoptotic activity upon TNF*α* treatment. One of the anti-apoptotic mechanisms of survivin-ΔEx3 is mediated by its association with both Bcl-2 and active caspase-3, in order to inhibit the activity of the bound enzyme (Figure 2, Figure 3). These data confirm our hypothesis that survivin-ΔEx3 is an anti-apoptotic factor, functioning like vIAP (Wang *et al*, 2002).

It is known that survivin-ΔEx3 is overexpressed in a number of tumours, and may contribute to tumourigenesis by protecting malignant cells from apoptosis by undefined mechanisms (Mahotka *et al*, 1999, 2002a, 2002b). Our data provide further insights into these mechanisms. Survivin-ΔEx3, like survivin, protects cells from methotrexate, lymphotoxin-*β* receptor, and CD95-induced apoptosis (You *et al*, 2006). Here, we showed that survivin-ΔEx3 also protects cell from TNF*α*-induced apoptosis, defining survivin-ΔEx3 as a key factor of the mitochondrial checkpoint of apoptosis.

In mammals, several adaptor proteins bridge caspases to Bcl-2 or Bcl-xL to prevent their activation. For example, Bap31 contributes to the regulation of procaspase-8, and this activity is dependent on the presence of Bcl-2 or Bcl-xL within the same complex (Ambrosini *et al*, 1997). A novel BH3-only protein, Spike, inhibits the formation of a complex between Bap31 and Bcl-xL, thereby favouring apoptosis (Mund *et al*, 2003). We previously showed that vIAP functions as an adaptor linking Bcl-2 to activated caspase-3, and thereby controlling its activity (Wang *et al*, 2002). Here, we showed that one of the anti-apoptotic mechanisms of survivin-ΔEx3 is also to act as an adaptor, linking Bcl-2 to active caspase-3, through its BH2 and BIR domains, respectively. This enables Bcl-2 to inhibit the activity of caspase-3 and to protect cells from apoptosis. Using a knock-down approach, we have demonstrated that survivin-ΔEx3 is essential to link Bcl-2 to caspase-3. Indeed, without any survivin-ΔEx3 expression detectable, we significantly reduced the quantity of caspase-3 interacting with Bcl-2 (Figure 2E). Survivin-ΔEx3 provides a link between two major apoptotic pathways. Our data were obtained in the context of TNF*α*-induced apoptosis, which is one of the main pathways involved in the control of cell survival–apoptosis balance.

We also studied the role of survivin-ΔEx3 upon Bax-induced apoptosis, which is part of the TNF*α*-induced responses. We showed that survivin-ΔEx3 is able to maintain the mitochondrial transmembrane potential and to prevent the translocation of cytochrome *c* from mitochondria (Figure 3C). This concurs with recent data showing that overexpression of survivin-ΔEx3 prevents LT*β*R-induced release of cytochrome *c* and Smac/DIABLO. LT*β*R is a member of another TNF superfamily group, which is specifically involved in developmental programmes (You *et al*, 2006).

Some members of the IAP family are E3 ubiquitin ligases (Vaux and Silke, 2005), involved in substrate ubiquitination and degradation by the 26S proteasome. For example, XIAP and livin protect cells from TRAIL-induced apoptosis by targeting pro-apoptotic molecules, such as Smac/DIABLO, for proteasomal degradation (MacFarlane *et al*, 2002; Ma *et al*, 2006). It remains to be investigated whether survivin-ΔEx3 holds ubiquitin-ligase activity, or interacts with an E3 ligase to protect cells from mitochondrial damage by targeting pro-apoptotic molecules for proteasomal degradation. In TNF*α*-induced responses, caspase-independent signalling pathways are also involved. We showed here that survivin-ΔEx3, upon TNF*α*-induced cell death, prevents the activation of caspase-3. As some IAPs regulate apoptosis by interacting with various components of the MAPK family (Zhang *et al*, 2003; You *et al*, 2006), survivin-ΔEx3 may also be able to play a role via caspase-independent pathways.

We have also shown that survivin-ΔEx3 is able to heterodimerise with survivin. This suggests that the splice variants of survivin contribute to regulate the balance between proliferation and cell death. As survivin links cell proliferation, survival, and stress responses, studies targeting survivin are being exploited, using diverse approaches from immunotherapy to small molecule antagonists (Altieri, 2006). Targeting survivin-ΔEx3, rather than survivin alone, may selectively and effectively destroy tumour cells, by suppressing their ability to resist apoptosis. Overall, these findings suggest that survivin-ΔEx3 is a potential target for future anti-cancer therapies.
