# Supplementary figures and images for: Characterisation of the anti-apoptotic function of survivin-ΔEx3 during TNFα−mediated cell death
Source: Br J Cancer. 2007 May 15;96(11):1659–66. doi: 10.1038/sj.bjc.6603768 (PMC2359927; doi:10.1038/sj.bjc.6603768)

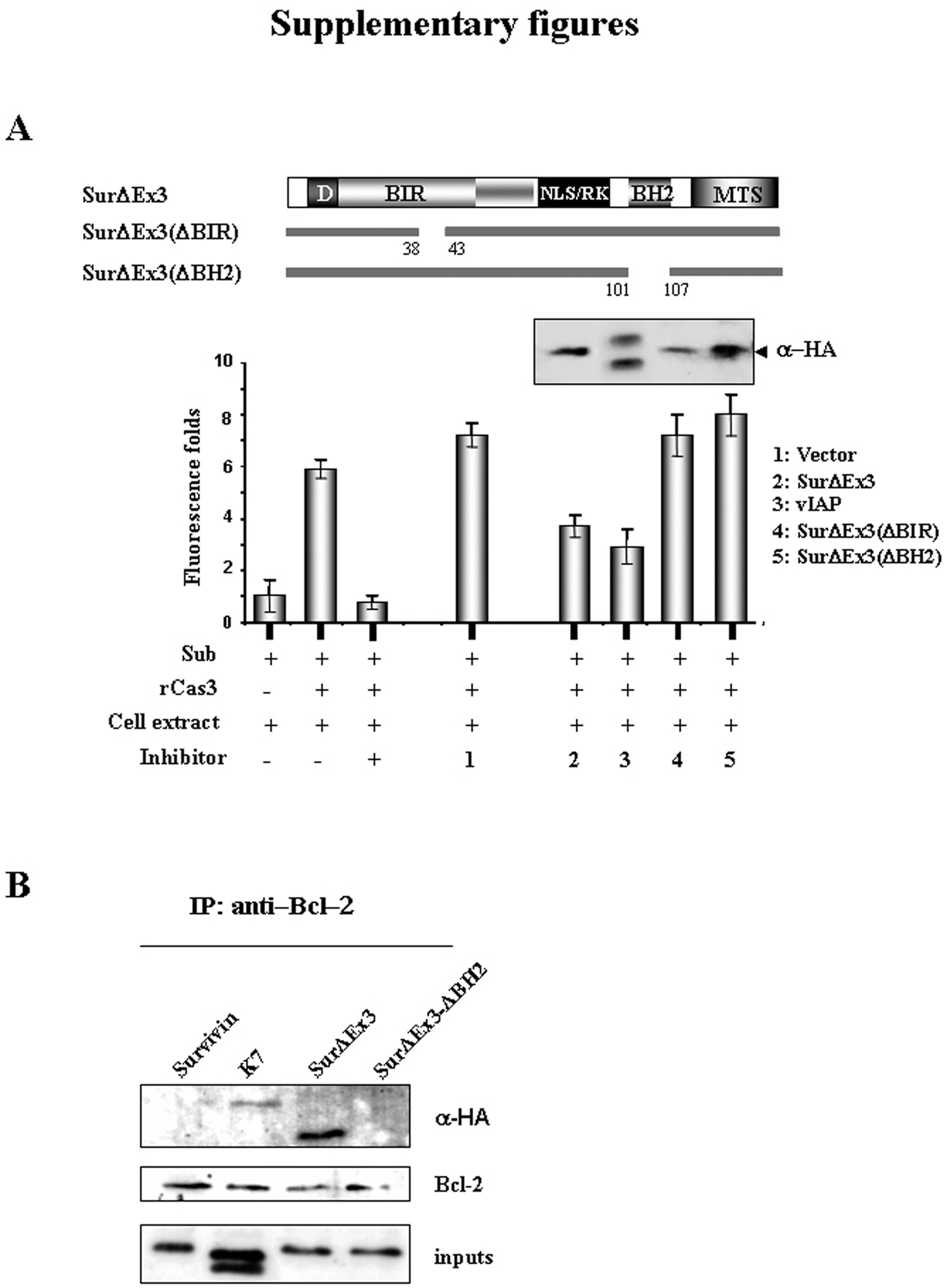

Supplement: Supplementary Figures [file 6603768x1.tif]
