# Supplementary material for: Characterisation of the anti-apoptotic function of survivin-ΔEx3 during TNFα−mediated cell death
Source: Br J Cancer. 2007 May 15;96(11):1659–66. doi: 10.1038/sj.bjc.6603768 (PMC2359927; doi:10.1038/sj.bjc.6603768)
Supplement: Supplementary Legend [file 6603768x2.doc]

**Supplementary figures:**

**A)** Requirement of both survivin-ΔEx3 BH2 and BIR domains for caspase-3-inhibition. Fluorescent substrate (Sub) and recombinant active caspase-3 (rCas3) were mixed with cellular extracts from cells transfected with the indicated plasmids. Reactions were incubated for 1 hr at 37°C, before monitoring fluorescence.

**B)** Survivin-ΔEx3 and K7, but not survivin binds to Bcl-2 *in vivo,* through its BH2 domain. Immunoprecipitations were performed in HeLa transfected with indicated plasmids.
